# NK Cell Phenotypic Modulation in Lung Cancer Environment

**DOI:** 10.1371/journal.pone.0109976

**Published:** 2014-10-09

**Authors:** Shi Jin, Yi Deng, Jun-Wei Hao, Yang Li, Bin Liu, Yan Yu, Fu-Dong Shi, Qing-Hua Zhou

**Affiliations:** 1 Tianjin Key Laboratory of Lung Cancer Metastasis and Tumor Micro-environment, Tianjin Lung Cancer Institute, Tianjin Medical University General Hospital, Tianjin, P.R. China; 2 Department of Medical Oncology, Harbin Medical University Cancer Hospital, Harbin, P.R. China; 3 Cancer Center, Research Institute of Surgery and Daping Hospital, Third Military Medical University, Chongqing, P.R. China; 4 Department of neurology, Tianjin Medical University General Hospital, Tianjin, P.R. China; The University of Hong Kong, China

## Abstract

**Background:**

Nature killer (NK) cells play an important role in anti-tumor immunotherapy. But it indicated that tumor cells impacted possibly on NK cell normal functions through some molecules mechanisms in tumor microenvironment.

**Materials and methods:**

Our study analyzed the change about NK cells surface markers (NK cells receptors) through immunofluorescence, flow cytometry and real-time PCR, the killed function from mouse spleen NK cell and human high/low lung cancer cell line by co-culture. Furthermore we certificated the above result on the lung cancer model of SCID mouse.

**Results:**

We showed that the infiltration of NK cells in tumor periphery was related with lung cancer patients' prognosis. And the number of NK cell infiltrating in lung cancer tissue is closely related to the pathological types, size of the primary cancer, smoking history and prognosis of the patients with lung cancer. The expression of NK cells inhibitor receptors increased remarkably in tumor micro-environment, in opposite, the expression of NK cells activated receptors decrease magnificently.

**Conclusions:**

The survival time of lung cancer patient was positively related to NK cell infiltration degree in lung cancer. Thus, the down-regulation of NKG2D, Ly49I and the up-regulation of NKG2A may indicate immune tolerance mechanism and facilitate metastasis in tumor environment. Our research will offer more theory for clinical strategy about tumor immunotherapy.

## Introduction

Lung cancer is one of the most common malignant tumors in the world, which has high morbidity and mortality and accounts for about 25.4% of all tumors. It has been an upward trend of the incidence rate in recent years [1]–[4]. The American Cancer Society released data show that 222,520 cases of respiratory cancer and 157,300 cases of death in 2010, which is in the first place of morbidity and mortality of all malignant tumors [5]. A clinical statistics of stage IV NSCLC in China showed that the 1-, 2-, 3-, 4- and 5-year survival rate was 44%, 22%, 13%, 9% and 6% respectively [6]. Currently, surgical resection is still the main method to prolong the survival time of lung cancer, but the invasion and metastasis of lung cancer is the biggest obstacle to improve the efficacy of the prognosis of lung cancer. For in-depth study of lung cancer malignant behavior and focus on comprehensive treatment of metastatic lung cancer, it is necessary to establish appropriate animal model to study lung cancer recurrence and metastasis and its comprehensive therapy.

Natural killer (NK) cell, also known as large granular lymphocytes, is an independent and non-specific immune cell. It has no MHC restriction to target cells recognition and destruction, and it can directly kill tumor cells and virus-infected target cells without antigen pre-sensitized [7], [8]. It also can produce a large number of immune-active cytokines to enhance or expand its anti-tumor effect, which can be regarded as the first line of the host defense system [9]. Several experimental evidences demonstrated the important role of NK cells in the elimination of tumor cells. Vivier et al report that a low NK cell cytotoxicity in peripheral blood was correlated with an increased cancer risk [10]. Furthermore, NK cells infiltrating in the tumor tissue was associated with good prognosis in colorectal [11], gastric [12], and lung [13] cancers.

With the development of tumor formation, malignant tumor cells and infiltrating immune cells interact and composed the tumor micro-environment. Most of studies published showed that a large number of immune cells infiltrating into tumor tissue played an important role in improving tumor prognosis [14], [15]. But as we all known, the prognosis of lung-associated malignancies is very terrible, even though there are many immune cells in the lung. We want to know if there is a differential composition of the immune cell infiltrate in malignant and non-malignant lung tissue areas, and even might potentially contribute to this effect. Esendagli G et.al found that in non-small cell lung cancer (NSCLC) patients, NK cells were not almost found in the malignant tissue regions, non-malignant counterparts were selectively populated by NK cells and those NK cells showed strong cytotoxic activity ex vivo [16].

So the impact of NK cell receptor expression and function may be different caused by the interaction between NK cells and tumor in the tumor micro-environment. By exploring NK cells in the body and/or lung cancer micro-environment, discuss its distribution, receptor expression, functional status with lung cancer invasion, metastasis and prognosis, clarify the mechanism of NK cells involved in lung cancer micro-environment from the cellular and molecular levels.

## Materials and Methods

### Tumor Samples and Ethics Statement

This study was conducted according to the principles expressed in the Declaration of Helsinki. All tumor samples used in this study were obtained from Tianjin Lung Cancer Institute and department of pathology in Tianjin Medical University General Hospital. All patients provided written informed consent for the collection of samples and subsequent analysis. And the study was approved by the Institutional Ethics Committee of Tianjin Medical University General Hospital.

### Paraffins Specimen and Frozen tissue origin of Lung cancer

Patients will be eligible for participation in the induction phase of the study if they have: histologic or cytologic diagnosis of NSCLC (including squamous carcinoma, adenocarcinoma and large cell carcinoma); no prior systemic chemotherapy, radiotherapy and biotherapy for lung cancer before surgery; no other cancer history; and ≤80 years of age.

Paraffins Specimen samples were collected from 84 patients diagnosed with lung cancer between January1st 2008 and January 31st 2011 (64 men and 20 women). The median age of patients was 60.7±7.9 years (range 40 to 78 years). There are different pathology types: 37 squamous carcinoma patients, 37 adenocarcinoma patients and 10 large cell carcinoma patients. At the time of diagnosis, patients were assessed according to the AJCC/UICC the sixth classification edition as follows: stage IIA-1 patients, stage IIB- 4 patients, stage IIIA-58 patients, stage IIIB-12 patients, stage IV-9 patients. The samples were applied from Tianjin Medical University Pathology Department.

Frozen tissue samples were generated from 66 surgical samples with lung cancer between January1st 2008 and January1st 2011. There are including 52 men and 14 women. The median age of patients was 60.5±8.4 years (range 40 to 78 years). There were including different pathology types: 28 squamous carcinoma patients, 28 adenocarcinoma patients and 10 large cell carcinoma patients. The patients were assessed according to the AJCC/UICC the sixth classification edition as follows: stage IIA-1 patients, stage IIB-4 patients, stage IIIA-45 patients, stage IIIB-9 patients, stage IV-7 patients. The samples were applied from Tianjin Lung Cancer Research Institute.

### Immunofluorescence

Immunophenotypes analysis of CD56 and CD16 were done as follow. In brief, the formalin-fixed, paraffin-embedded sections (4 µm) were warmed by ovenware for 45 min, then deparaffinized in xylene and rehydrated in a graded series of ethanol solutions. The sections were subsequently submerged in citric-sodium citrate buffer solution (pH 7) and microwave at high fire for 5 min, low fire for 15 min, and natural cooling to room temperature to retrieve the antigenicity. Endogenous peroxidase was quenched with 3% H_2_O_2_ for 30 min. After washing with PBS, the sections were incubated with 2% BSA for 2 hours in room temperature and then incubated with CD56 and CD16 antibody (Santa Cruz, diluted at 1∶100) overnight at 4°C. The sections were incubated with mouse anti-goat and goat anti-mouse fluorescence antibody (Santa Cruz, diluted at 1∶200) for 35 min and the DAPI for 5 min. After washing with PBS fourth times, the sections were mounting by glycerol (PH 9.0). The red fluorescence expressed CD56^+^, the green fluorescence expressed CD16^+^, the blue fluorescence expressed cell nucleus. We can find the CD56^+^ and/or CD16^+^ expressing in the NK cell. The SPOT image analysis software was used in the analysis process. The percentage of CD56 and/or CD16 positive cells was determined by counting per section. The NK cell count of per view field was scored according to the following criteria: +, the NK cell counts of per view field<10; ++, the NK cell counts of per view field>10, <20; +++, the NK cell counts of per view field>20. All fields in per section in three independent experiments were calculated.

### RNA extraction, cDNA synthesis, and DNA isolation

The total RNA was extracted with the Trizol reagent (Invitrogen, Carlsbad, CA) according to the manufacturer's protocol. The total RNA was digested with R Nase-free D Nase-I (Promega, Madison, USA) and used for cDNA synthesis with the reverse transcriptase system (Takara Biotech, Dalian, China).

### Quantitative real-time PCR

The cDNA synthesis was performed as described above. The specific primer of RT experiment were as follow: NK1.1:5′- TCTCTTGAATAAACACACAGCAT -3′, NKG2A: 5′- CTGAGAAGGATTTTG -3′, NKG2D: 5′- TTCTCACAGTTCCTCT -3′, LY49I: 5′- TTCTATTCTTGCTTTAG -3′. The primer pairs and GAPDH primer pairs were used for Q-PCR with the SYBR Premix Ex Taq RT-PCR Kit (TaKaRa, Dalian, China) in a ABI PRISM 7900 Real-time System (Bio-Rad) with the following conditions: 95°C for 30 s, 40 amplification cycles (95°C for 5 s and 60°C for 30 s) and a melting cycle from 60°C to 95°C. The data was analyzed using the ABI PRISM 7900 Manager software. All reactions were performed with three technical replicates.

### Cell

The high and low metastasis large cell lung cancer cell lines L9981-Luc and NL9980-Luc from Tianjin Lung Cancer Institute [17], [18] were maintained in RPMI 1640 medium (Hyclone, America) containing 10% fetal bovine serum (FBS), 100 U/ml penicillin and 100 µg/ml streptomycin (Hyclone, America) in a fully humidified incubator (Nuaire, US Autoflow) at 37°C with 5% CO_2_. The cells were kept in an exponential growth phase during experiments.

### SCID model

SCID (severe combined immune deficiency) mice purchased from Chinese academy of medical sciences animal institutions were housed in pathogen-free animal facilities. Female mice used were 5–7 wk of age at the experiment's inception. Female SCID mice (16 animals for each experimental group) were inoculated subcutaneously into the right inguen with 2*10^6^ cells suspended in 150 ul of PBS. Nine animals in control group had no inoculation. Tumor sizes were measured every 7 days and their fluorescence intensity were measured with America's true essence of living imaging system (XENOGEN IVIS200). After 6 weeks of observation, dissection was performed and explanted tumors, lungs and spleen were removed for the further analysis. These experiments were repeated at least twice to confirm the results. The animal experimental protocols were approved by the Committee for Ethics of Animal Experimentation and the experiments were conducted in accordance with the Guidelines for Animal Experiments in Tianjin Medical University Cancer Research Center.

### Lymphocyte collect and Flow Cytometry detect

The lungs and spleens were polished into pieces. Then the lymphocytes collect were extracted with the Lymphocyte Separation Medium (Shenzhen, China) according to the manufacturer's protocol. Percentages of NK cells were evaluated and separated with flow cytometry using monoclonal antibodies (MoAbs) anti-CD3^−^ FITC/NK1.1^+^ PE (BD Phamingen, San Di ego, CA). During analysis, the CD3^−^/NK1.1^+^ Population was determined. To determine the surface expression of the NK cell ligands on NK cells, the cells were then stained with the goat anti-mouse NK1.1-FITC, CD3- Alexa Fluor 647, NKG2A-FITC, NKG2D-PE, Ly49I-PE (BD Phamingen, San Di ego, CA) for 30 min at 37°C in the dark. The analysis was performed on the FACS Sort (Becton Dickinson, Mountain View, CA) using Cell Quest software (Becton Dickinson) and the cell surface expression was quantified by the value of the mean fluorescence intensities obtained with the specific mAbs.

### Co-culture

Purified NK1.1^+^/CD3^−^ NK cells (activated with 100U/ml IL-2 during 2 h) from mouse spleen were co-cultured with lung cancer cell line L9981-Luc/NL9980-Luc as (A)0.25∶1, (B)0.5∶1, (C)1∶1 and (D)2∶1 for 24, 48, 72 hours. After co-culture, the fluorescence intensity of NK cells was measured with America's true essence of living imaging system.

### Quantitative real-time PCR

RNA was isolated from explanted tumors via Trizol and reverse-transcribed using special priming (Invitrogen). Quantitative RT-PCR was performed on an ABI PRISM 7900 instrument, using SYBR green assays. The specific primers are listed in the following (Table 1). The amplification was performed as follows. After initial denaturation step at 95°C for 30 seconds, templates were denatured at 95°C for 5 seconds, primers were annealed and DNA extension was performed at 60°C for 30 seconds (each cycle was repeated 40 times). The expression of target genes was compensated using the expression of GAPDH and presented by the expression ratio between untreated control cells and treated cells.

**Table 1:** NK cells phenotype expressed in different pathological type of lung cancer.

Pathological type	NK cellular infiltration extent			Total	χ*^2^*	*P* value
	Grade1	Grade2	Grade3			
CD56	37(44.0%)	19(22.6%)	28(33.3%)	84(100.0%)	0.126	0.998
CD16	38(45.2%)	19(22.6%)	27(32.1%)	84(100.0%)		
CD56CD16	38(45.2%)	20(23.8%)	26(31.0%)	84(100.0%)		

### Statistical analysis

Experiments were performed three times. The Data are presented as mean values ± SD. The statistical evaluation was performed using one-way ANOVA tests or rank-sum test when measurement data is Gaussian distribution with the SPSS Software system, version 17.0. If the data is enumeration data, Chi-square test was used. *P* values of less than 0.05 were considered statistically significant. Overall survival rate was compared by Kaplan-Meier survivorship curve, Log-rank rank-sum test used among groups.

## Results

### NK cell localization and morphology characteristic in lung cancer micro-environment

**Figure 1:**
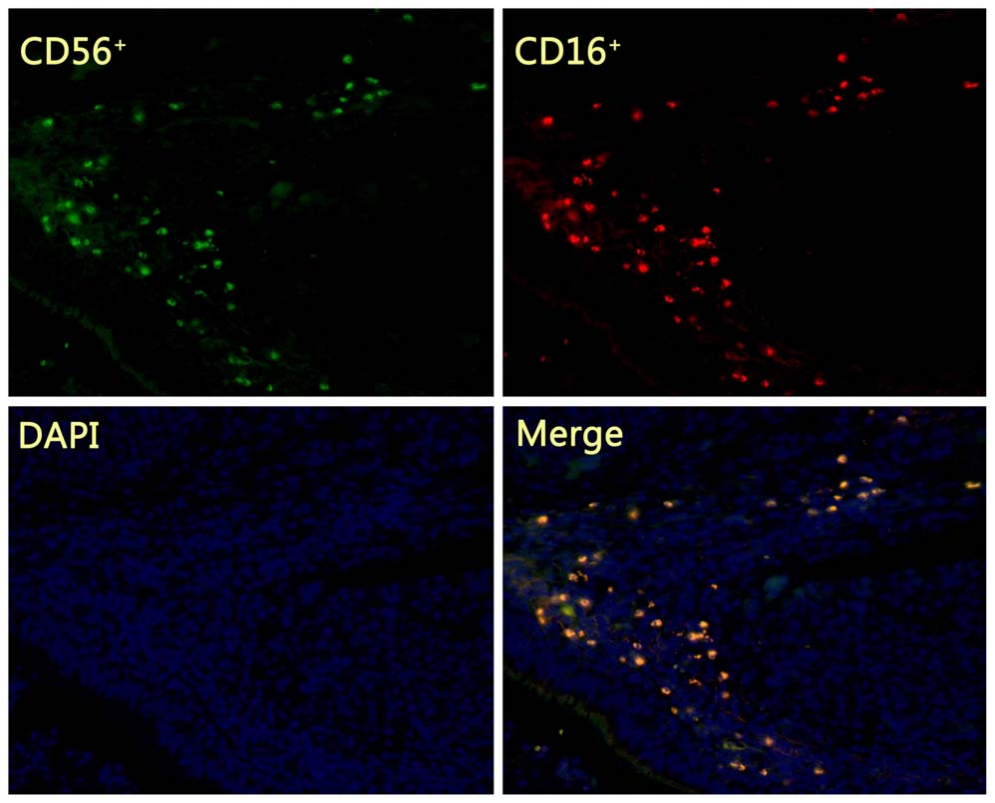
Immune fluorescence detecting the phenotype of NK cells in lung cancer tissues. The infiltrated NK cells in the lung cancer tissue were mainly concentrated in the tumor stroma. NK cells shows with CD56 single positive (green fluorescence), CD16 single positive (red fluorescence) and CD56CD16 double positive (yellow fluorescence) in the lung cancer stroma tissue.

The infiltrated NK cells in the lung cancer tissue were mainly concentrated in the tumor stroma, constituting the tumor micro-environment (Fig.1). In NSCLC tissues, CD56^+^ NK cells with 1, 2 and 3 degree of infiltration were 44.0%, 22.6%, 33.3%, CD16^+^ NK cells were 45.2%, 22.6%, 32.1%, and CD56^+^CD16^+^ NK cells were 45.2%, 23.8%, 31.0%, respectively. No significant difference was observed (*p*>0.05) among NK cells with CD56 single positive, CD16 single positive and CD56CD16 double positive in the lung cancer tissue (Table 1).

### NK cell infiltration and lung cancer patients clinical pathology physiological feature

**Figure 2:**
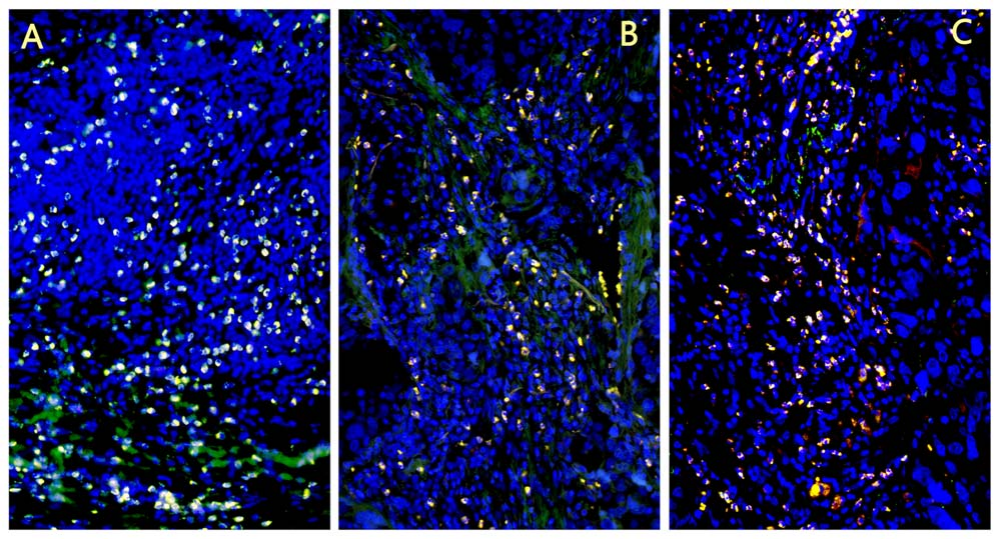
CD56^+^CD16^+^ NK cell infiltration extent in different pathological type of lung cancer. A. Lung squamous carcinoma; B. Lung adenocarcinoma; C. Large cell lung cancer. The expression of NK cells in squamous cell carcinoma was significantly higher than in adenocarcinoma and large cell carcinoma.

The 1, 2 and 3 degree with CD56^+^CD16^+^ NK cell infiltration were 24.3%, 32.4%, 43.2% in squamous cell carcinomas, and 62.2%, 16.2%, 21.6% in adenocarcinoma and 50.0%, 10.0%, 40.0% in large cell lung cancer, respectively. There was significant difference of CD56^+^CD16^+^ NK cell infiltration degree between different pathological types of lung cancer (*p* = 0.019) (Fig.2, Table 2). The expression of NK cells in squamous cell carcinoma was significantly higher than in adenocarcinoma and large cell carcinoma. The 1, 2 and 3 degree with NK cell infiltration were 48.1%, 3.7%, 48.1% in lung cancer patients with no history of smoking, were 42.1%, 31.7% and 26.3% in patients with smoking history. Smoking history of lung cancer patient is related to NK cell infiltration degree (*p* = 0.011). The 1, 2 and 3 degree with NK cell infiltration were 60.6%, 18.4%, 21.1% in the T1-T2 cancer, and 42.1%, 31.7%, 43.5% in the T3-T4 cancer, respectively. A significant difference of NK cell infiltration degree was found between different size of the tumor (*p* = 0.019) (Table 3).

**Table 2:** CD56^+^CD16^+^NK cells infiltration extent in different pathological type of lung cancer.

Pathological type	CD56^+^CD16^+^NK cellular infiltration extent			Total	χ*^2^*	*p* value
	Grade1	Grade2	Grade3			
squamous cell carcinoma	9(24.3%)	12(32.4%)	16(43.2%)	37(100.0%)	11.800	0.019*
adenocarcinoma	23(62.2%)	6(16.2%)	8(21.6%)	37(100.0%)		
Large cell cancer	5(50.0%)	1(10.0%)	4(40.0%)	10(100.0%)		
Total	37(44%)	19(22.6%)	28(33.3%)	84(100.0%)		

*p<0.05, Difference have statistical significance.

**Table 3:** The clinical features of lung cancer patients and NK cell infiltration extent.

Clinical features	Cases	CD56^+^CD16^+^NK cellular infiltration extent			χ^2^	*p* value
		Grade1	Grade2	Grade3		
**Sex**						
Male	64	29(45.3%)	16(25.0%)	19(29.7%)	1.843	0.398
Female	20	8(40.0%)	3(15.0%)	9(45.0%)		
**Age**						
≤60	39	18(46.2%)	7(17.9%)	14(35.9%)	0.919	0.632
>60	45	19(42.2%)	12(26.7%)	14(31.1%)		
**Smoking history**						
no	27	13(48.1%)	1(3.7%)	13(48.1%)	9.066	0.011*
have	57	24(42.1%)	18(31.7%)	15(26.3%)		
**Primary tumor(T1-4)**						
T1-T2	38	23(60.5%)	7(18.4%)	8(21.1%)	7.958	0.019*
T3-T4	46	14(30.4%)	12(26.1%)	20(43.5%)		
**Regional lymph node (N0-3)**						
N0-N1	17	4(23.5%)	7(41.2%)	6(35.5%)	5.307	0.070
N2-N3	67	33(49.3%)	12(17.9%)	22(32.8%)		
**Metastasis (M0-1)**						
M0	75	34(45.3%)	18(24.0%)	23(30.7%)	2.346	0.309
M1	9	3(33.3%)	1(11.1%)	5(55.6%)		
Total	84	37(44%)	19(22.6%)	28(33.3%)		

*p<0.05, Difference have statistical significance.

### NK cell infiltration and lung cancer patients prognosis

**Figure 3:**
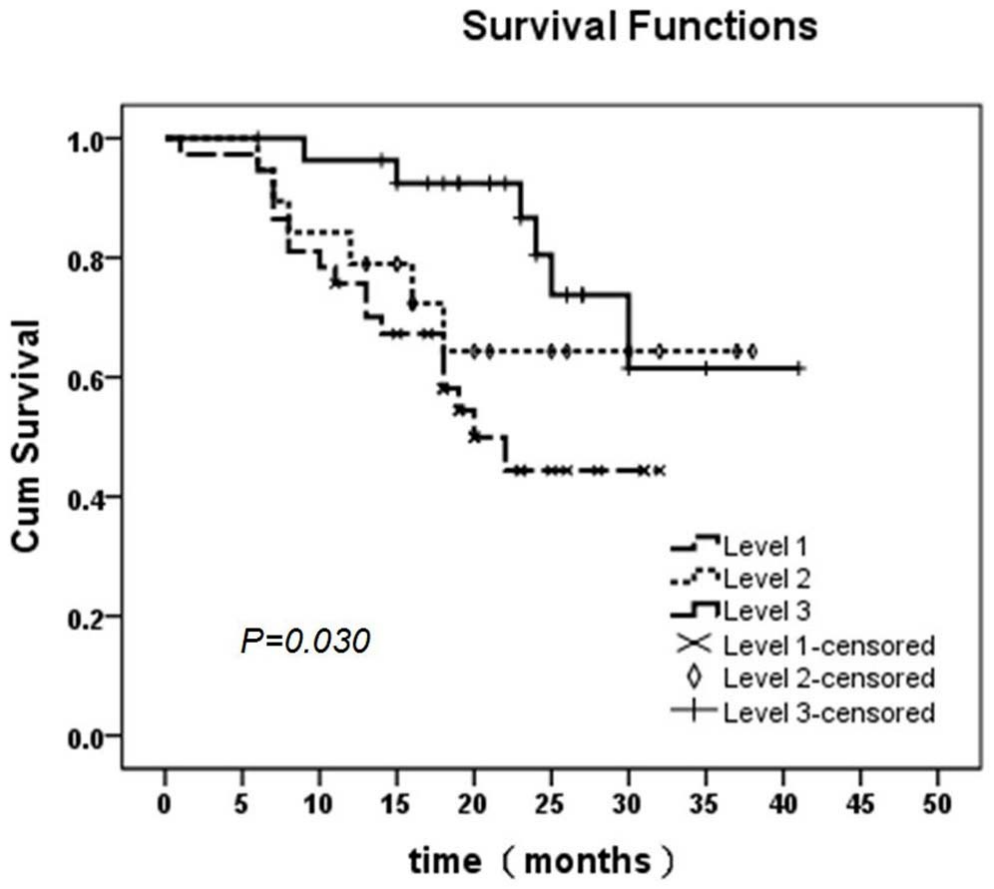
The survival time of different NK cell infiltration extent in the lung cancer. The survival time of lung cancer patient was positively related to NK cell infiltration degree in lung cancer. The more infiltration of NK cells were existed, the longer the survival time of patients did. The patients in level 3 group have the longest survival time. Among three groups statistical significance are exit. (*p* = 0.030)

The survival time of lung cancer patient was positively related to NK cell infiltration degree in lung cancer. The more infiltration of NK cells were existed, the longer the survival time of patients did (*p* = 0.030) (Fig.3, Table 4).

**Table 4:** NK cells infiltrating extent in the lung cancer and overall survival time.

NK cell	Survival Time(months)				Chi-Square	*p* value
	<12 (m)	13∼24(m)	>25 (m)	Overall		
Level 1	10(27.0%)	21(56.8%)	6(16.2%)	37	7.017	0.030*
Level 2	4(21.1%)	9(47.4%)	6(31.6%)	19		
Level 3	2(7.1%)	14(50.0%)	12(42.9%)	28		
Overall	16(19.0%)	44(52.4%)	24(28.6%)	84		

*p<0.05, Difference have statistical significance.

### Real-time PCR confirm NK cell infiltration extent in lung cancer

**Figure 4:**
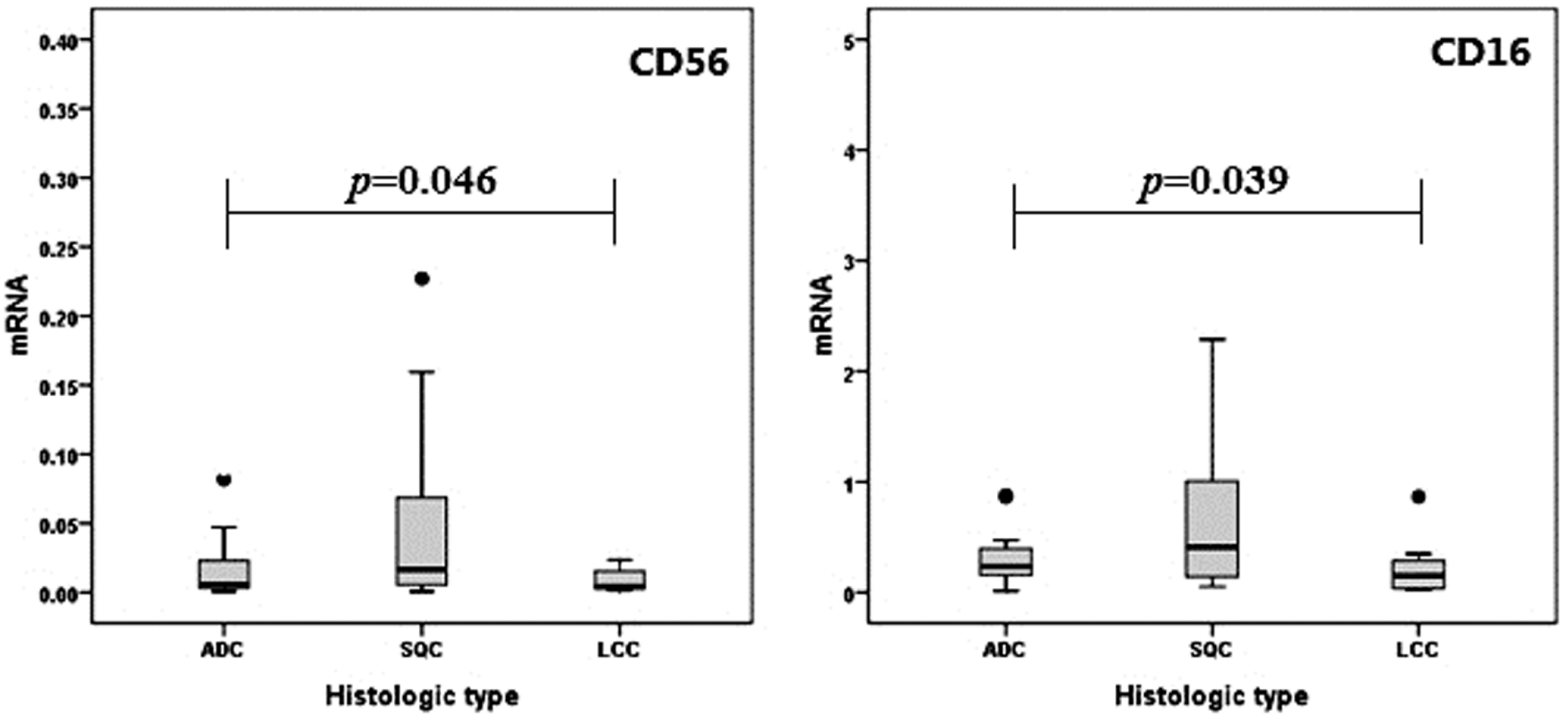
NK cells phenotype mRNA expression levels in different histologic type of lung cancer. NK cells receptors CD56 and CD16 phenotype mRNA in different histologic types of lung cancer have statistical significance.

The differences of NK cell receptors CD56 and CD16 mRNA expression between different pathological types of lung cancer was detected and verified by Real-time PCR. We found that NK cells phenotype mRNA in different histologic type of lung cancer have statistical significance (Fig.4). This is in according with the result of the histology.

### Transplanted tumors-lung metastasis models established

**Figure 5:**
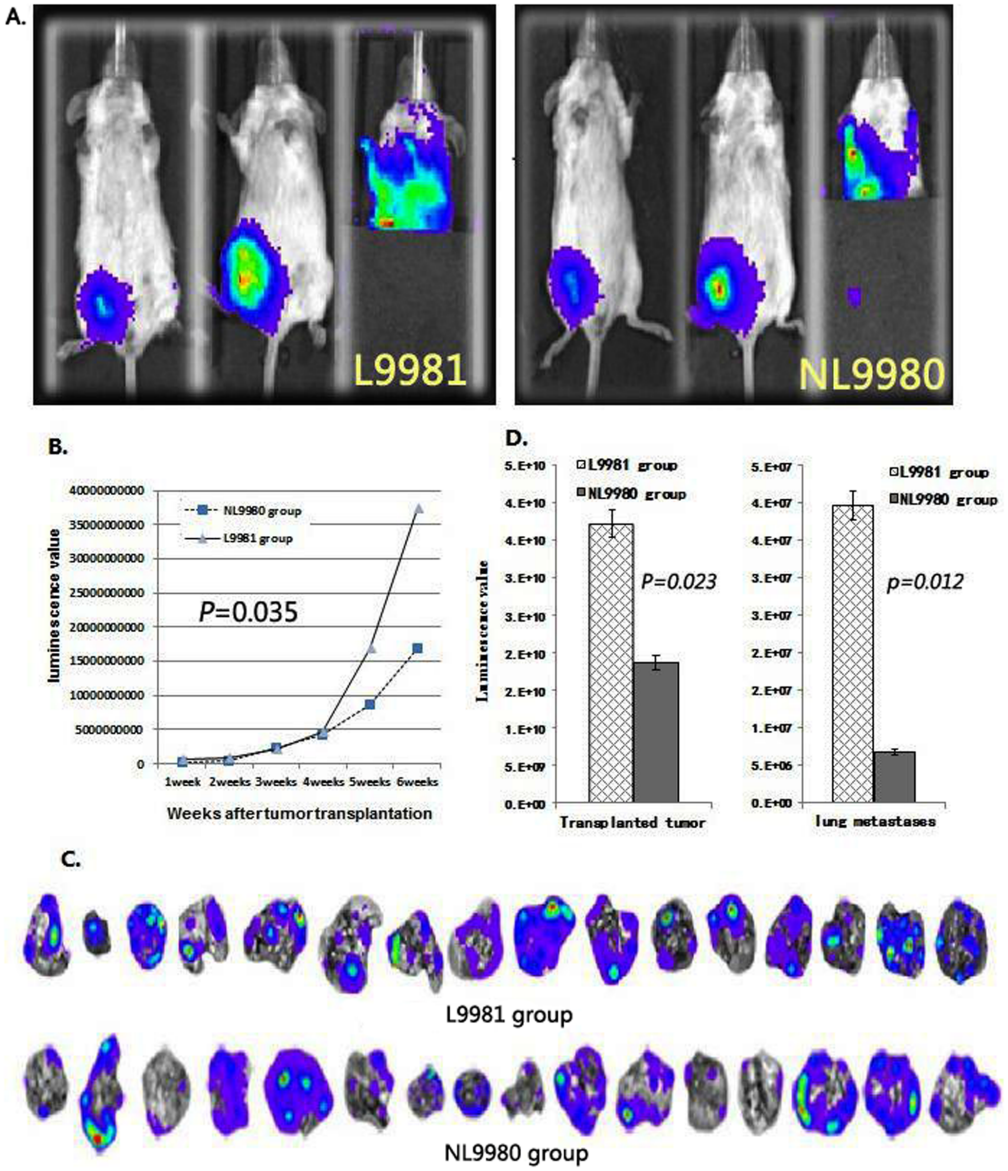
Transplanted tumors-lung metastasis models of high (L9981)/low (NL9980) metastatic human large cell lung cancer cell lines. A: Living fluorescence imaging detect mouse model (the left is the right inguen subcutaneously tumor for 1 week and the middle is for 6 weeks after injection, the right is the lung metastases tumors in six weeks after inoculation.), B: Growth curve of subcutaneously transplant tumor in SCID mouse, C: lung metastases luminescence imaging of tumor-bearing mouse in vitro, D: The comparison of luminescence value between transplanted tumor and lung metastases of high (L9981)/low (NL9980) metastatic human large cell lung cancer cell lines.

Transplanted tumors-lung metastasis models were successful established in SCID mouse with high (L9981)/low (NL9980) metastatic human large cell lung cancer cell lines. The distant metastasis of the xenograft tumor was detected by fluorescence imaging in vitro (Fig.5A). Compared with low-metastatic group (6.84×10^6^±3.26×10^6^), the lung metastases fluorescence value of mice in the high-metastatic group (30.97×10^6^±14.3×10^6^) was remarkably higher than that in low-metastatic group (*p* = 0.035) (Fig.5B, C, D).

### NK cell phenotypic modulation in lung cancer micro-environment

**Figure 6:**
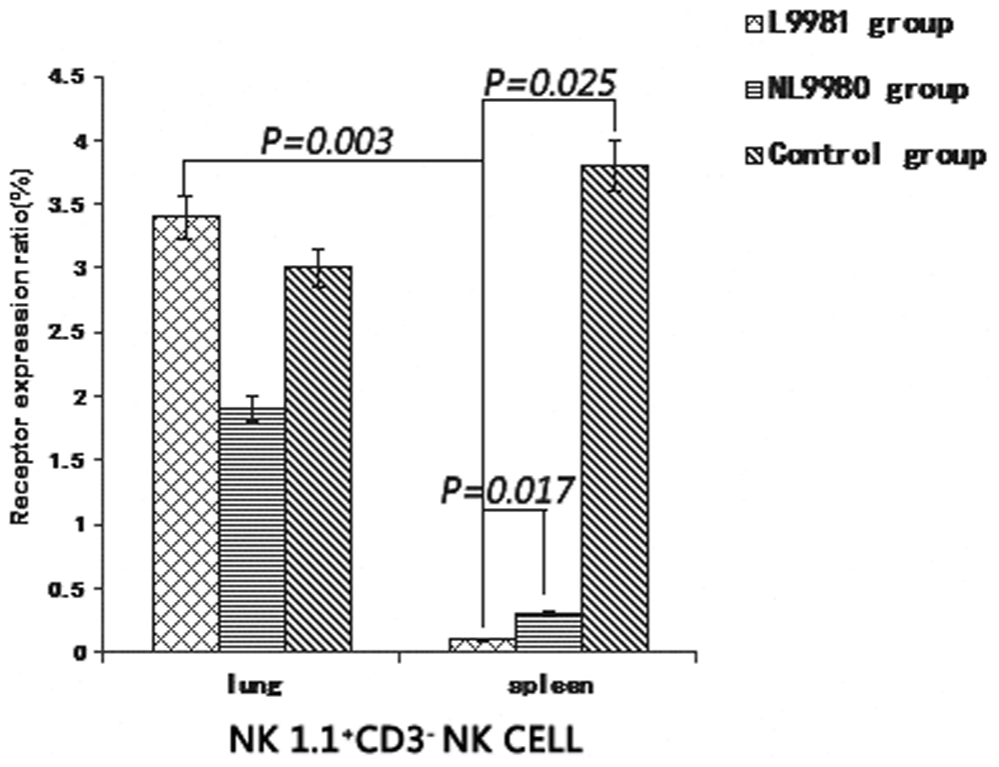
NK1.1^+^ CD3^−^ NK cell receptor expression in lung and spleen of different groups. NK1.1^+^CD3^−^ NK cells in lungs of high-metastatic group were significantly higher than that in spleen (*p* = 0.003). NK1.1^+^CD3^−^ NK cells in spleen of high-metastasis group (0.10±.06) were significantly lower than that in low-metastasis group (*p* = 0.017) and control group (*p* = 0.025).

NK1.1^+^CD3^−^ NK cells in lungs of high-metastatic group (3.40±0.90) were significantly higher than that in spleen (0.10±0.06) (*p* = 0.003). NK1.1^+^CD3^−^ NK cells in spleen of high-metastasis group (0.10±0.06) were significantly lower than that in low-metastasis group (1.66±0.82) and control group (3.80±2.05) (*p* = 0.017, *p* = 0.025) (Fig.6).

**Figure 7:**
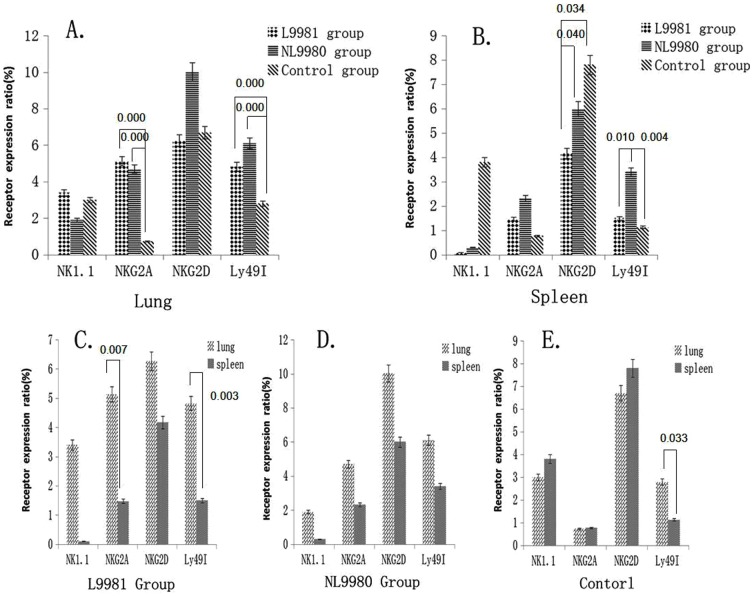
NK cell phenotypic modulation in lung cancer micro-environment. **A**: NKG2A expression level of NK cells from lung in high-metastatic L9981 group were significantly higher than that in low-metastatic NL9980 group and control group (*p* = 0.000). Ly49I expression level from the lung in L9981 group and NL9980 group was significantly higher than that in control group (*p* = 0.000) **B**: NKG2D expression level of NK cells from spleen in L9981 group (4.17±0.85) were remarkably lower than that in control group (*p* = 0.034) and NL9980 group (*p* = 0.040). Ly49I expression level in NL9980 group was remarkably higher than that in L9981 group (*p* = 0.010) and control group (*p* = 0.004). **C**: NKG2A expression level of NK cells from lung was significantly higher than that from spleen in high-metastatic L9981 group (*p* = 0.007) and Ly49I expression level from lung was remarkably up-regulated than that in spleen (*p* = 0.003) **D**: No significant difference of all the receptors expression level of NK cells was existed in low metastasis NL9980 group. **E**: The Ly49I expression level of NK cells from the lung was significantly higher than that from the spleen in control group (*p* = 0.033).

NKG2D expression level of NK cells from spleen in high-metastasis group (4.17±0.85) were remarkably lower than that in control group (7.80±2.67) (*p* = 0.034) and low-metastasis group (6.00±0.96) (*p* = 0.040) (Fig.7B).

NKG2A expression level of NK cells from lung (5.13±2.36) was significantly higher than that from spleen (1.47±0.68) in high-metastatic group (*p* = 0.007) (Fig.7C). NKG2A expression level of NK cells from lung in high-metastatic group (5.13±2.36) were significantly higher than that in low-metastatic group (4.70±1.96) and control group (0.73±0.26) (*p* = 0.000) (Fig.7A).

Ly49I expression level of NK cells from lung in high-metastatic group (4.82±1.78) was remarkably up-regulated than that in spleen (1.50±0.10) (*p* = 0.003) (Fig.7C). The Ly49I expression level of NK cells from the lung (2.79±0.40) was significantly higher than that from the spleen (1.13±0.40) in control group (*p* = 0.033) (Fig.7E). Ly49I expression level of the NK cells from the lung in high-metastatic group (4.82±1.78) and low-metastatic group (6.11±2.23) was significantly higher than that in control group (2.79±0.40) (*p* = 0.000) (Fig.7A). Ly49I expression level of NK cells from the spleen in low-metastatic group (3.40±1.00) was remarkably higher than that in high-metastatic group (1.50±0.10) (*p* = 0.010) and control group (1.13±0.40) (*p* = 0.004) (Fig.7B).

### NK cell lethal effect in lung cancer micro-environment

NK1.1^+^CD3^−^ NK cells activated by IL-2 in vitro have significant cytotoxic activity to high/low metastatic lung cancer large cells. A significant difference was observed between different effector-target ratio (*p* = 0.005, *p* = 0.017). NK cells had higher cytotoxic activity to NL9980 than that to L9981 in the same effector-target ratio (*p* = 0.035) (Table 5).

**Table 5:** Detection of NK cell cytotoxicity by luminescence *in vitro*.

Group	Time (h)	Luminescence value(E+05)					*p* value	
		0.25∶1**	0.5∶1**	1∶1**	2∶1**	Control		
L9981 group	24	181±49.7	160±20.3	123±11.7	99±7.2	221±18.7	0.005*	0.035*
	48	174±19.0	150±35.4	105±10.7	67±7.6	224±26.7		
	72	185±18.9	149±35.2	102±11.2	64±12.6	246±26.9		
NL9980 group	24	131±46.7	122±19.0	113±5.4	11±2.9	123±10.7	0.017*	
	48	124±14.1	119±11.4	113±8.4	18±5.6	125±15.9		
	72	132±14.0	123±10.7	102±8.9	14±5.8	142±17.0		

*p<0.05, Difference have statistical significance.

**NK cell: lung cancer cell.

### NK cell receptor mRNA expressing in subcutaneously transplanted tumor of mouse

**Figure 8:**
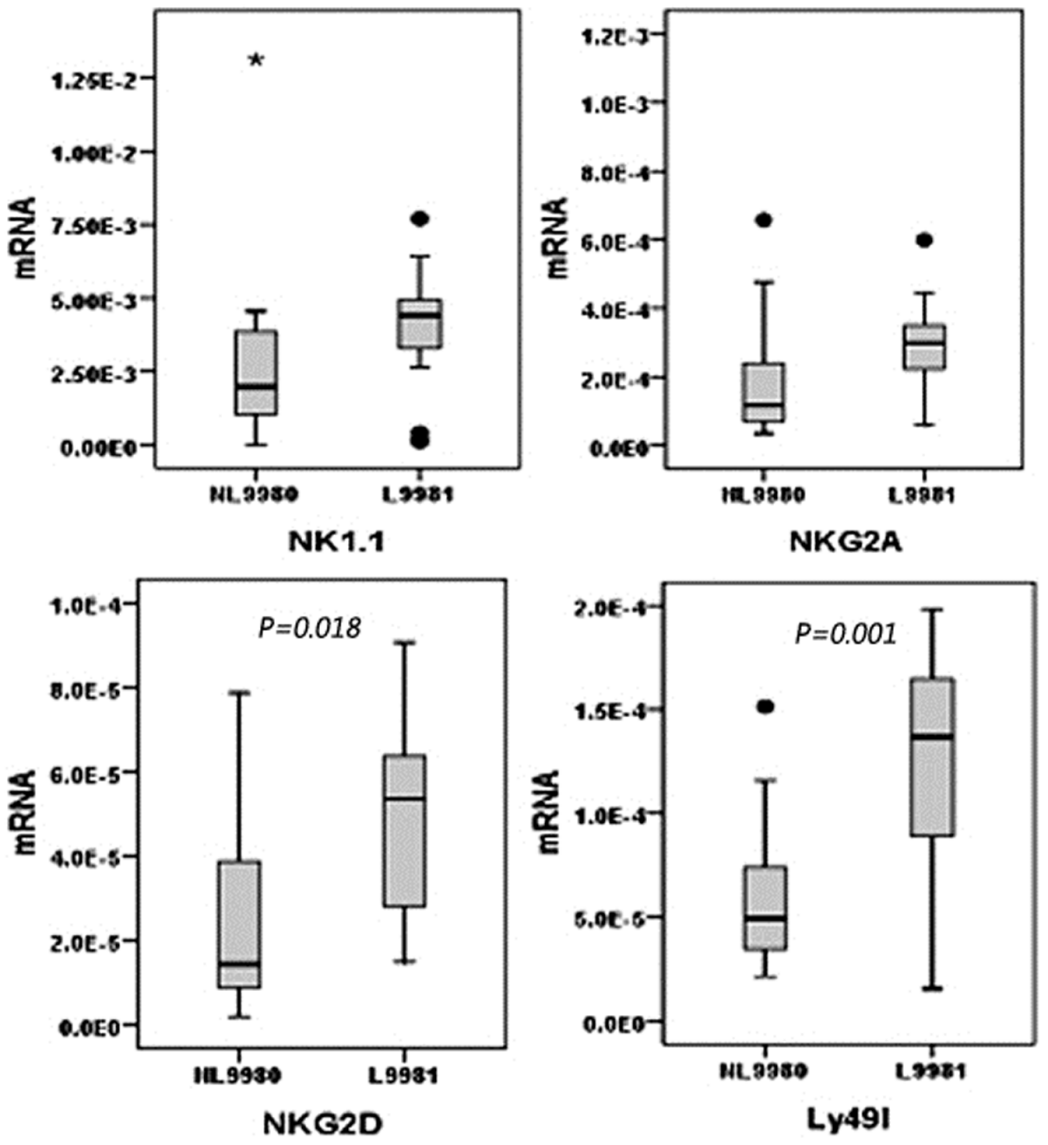
NK cell different receptors mRNA expressing in subcutaneously transplanted tumor of mouse. NKG2D expression level of NK cells in high-metastasis L9981 group was significantly higher than that in low-metastasis NL9980 group (*p* = 0.018), and the Ly49I expression level of NK cells in L9981 group was also remarkably higher than NL9980 group (*p* = 0.001). No significant difference of the NK1.1 and NKG2A expression level of NK cells was existed between high and low metastasis group.

In the transplanted tumors of tumor-bearing group, NKG2D expression level of NK cells in high-metastasis group was significantly higher than that in low-metastasis group (*p* = 0.018), and the Ly49I expression level of NK cells in high-metastasis group was also remarkably higher than low-metastasis group (*p* = 0.001). However, no significant difference of the NK1.1 and NKG2A expression level of NK cells was existed between high and low metastasis group (*p*>0.05) (Fig.8).

## Discussion

This study revealed that NK cells infiltration and NK cells receptor expression change in NSCLC tumor environment. From our research, we found that the NK cells infiltrated mainly in the tumor stroma, constituting the tumor micro-environment. These observations are in agreement with previous observations demonstrating that lung tumor micro-environment [16]. In addition, we got the surprise that the number of NK cell infiltrating in lung cancer tissue is closely related to the pathological types, size of the primary cancer, smoking history and prognosis of the patients with lung cancer.

Previous literature implied that some molecules expressing in the tumor and some mediums released from tumor commonly led to tumor escape mechanisms from NK cell immunological surveillance [19], [20]. There are high levels of non-classical MHC I molecular HLA - E and HLA - G in the tumor cell surface. They were NK cell activation CD94/receptor NKG2A and cell surface immune globulin sample transcription molecular 2 (ILT2) inhibitory ligand, which can restrain NK cell damage function [21]–[23]. At the same time, tumor cells can secrete some tumor suppressor factors inhibition of NK cell function, such as IL-10 [24] and TGF-β [25]. Surrounding the tumor infiltrating NK cell, some special changes in some other human tumors had been researched. In kidney cancer, the NK cell within tumor only was activated by IL-2 stimulation could have the target cell solution function. And to the same patients there were significant differences between the peripheral blood NK cell phenotype with in tumor [26]–[28]. The NK cell surface receptor DNAM-1, 2B4, CD16 expression reduced in ovarian cancer, which can result in the NK cell activation function were badly damaged [29].

Our research result prompt that the activated receptor of NK cells is down-regulated and inhibitory receptor of NK cells is up-regulated in the transplanted tumor and the distant metastatic lesions of human large cell lung cancer cell lines, which might be the main reason leading to NK cell killing ability decreased.

NKG2D is a receptor for MHC class I chain-related A and B molecules, which are frequently expressed by epithelial cancers. Ligation of NKG2D induces anti-tumoral effector functions in both NK and T cells. NKG2D recognition plays an important role in tumor immune surveillance [30] and that NKG2D primarily acts to trigger perforin-mediated apoptosis [31]. Our research verified that NKG2D expression level of NK cells from spleen in high-metastasis group were remarkably lower than that in control group (*p* = 0.034) and low-metastasis group (*p* = 0.040). In the transplanted tumors of tumor-bearing group, NKG2D mRNA expression level of NK cells in high-metastasis group was significantly higher than that in low-metastasis group (*p* = 0.018).

NKG2A is an inhibitory NK receptor that has been reported to form disulfide-linked heterodimers with invariant CD94. The NKG2A ligand has been identified as HLA-E, a non-classical MHC class-I b molecule that is widely distributed among various tissues, exhibits relatively low surface expression, and has limited polymorphism [32]. The inhibitory receptor CD94/NKG2A plays a major role in NK cell-mediated lysis of activated CD4^+^T cells [33]. NKG2A expression level of NK cell from lung was significantly higher than that from spleen in high-metastatic group (*p* = 0.007). NKG2A expression level of NK cells from lung in high-metastatic group were significantly higher than that in low-metastatic group and control group (*p* = 0.000). Thus, the down-regulation of NKG2D and the up-regulation of NKG2A may indicate immune tolerance mechanism and facilitate metastasis in tumor environment.

Interaction of MHC class I with the Ly49 receptors prevent the activation of NK cells and thereby the lysis of the target cell. Thus, NK cells utilize the Ly49 receptors to differentiate ‘self’ from ‘missing-self ’. Loss of MHC class I molecules on target cells relieves the NK cell of Ly49-mediated inhibition, thus allowing the NK cells to mediate cytotoxicity [34]. The Ly49I expression level of NK cells from the lung was significantly higher than that from the spleen in control group (*p* = 0.033). Ly49I expression level of the NK cells from the lung in high-metastatic group and low-metastatic group was significantly higher than that in control group (*p* = 0.000). In the transplanted tumors of tumor-bearing group, the Ly49I mRNA expression level of NK cells in high-metastasis group was also remarkably higher than low-metastasis group (*p* = 0.001).

Furthermore, a higher resistant to cytotoxic activity of NK cell exist in the human high-metastatic large cell lung cancer cell line L9981, which is much higher than that in the low-metastatic large cell lung cancer cell line NL9980. In addition, it is possible that other receptors of NK cells changed in tumor environment. In melanoma, NK cells could significantly increase the expression of CD86, and ligation of CD86 with CTLA4Ig significantly increased the ability of NK cells to kill tumor cells [35]. In two spontaneous metastasis models, the B16F10.9 melanoma (B16) and the Lewis lung carcinoma (D122) in the NCR1 knockout mouse, NKp46/NCR1 is directly involved in the killing of B16 and D122 cells in vitro and plays an important role in controlling B16 and D122 metastasis in vivo [36].

So, the variation of NK cell receptors might be useful in the prediction of tumor cell invasion and metastasis capacity, and further studies are needed to figure out the exact mechanism.

## References

[1] Jemal A, Murray T, Ward E, Samuels A, Tiwari RC, et al (2005) Cancer statistics, 2005. CA Cancer J Clin 55: 10–30. 15661684 10.3322/canjclin.55.1.10

[2] Yoshimi I, Sobue T (2004) International comparison in cancer statistics: Eastern Asia. Jpn J Clin Oncol 34: 759–763. 15640509 10.1093/jjco/hyh144

[3] Parkin DM, Bray F, Ferlay J, Pisani P (2005) Global cancer statistics, 2002. CA Cancer J Clin 55: 74–108. 15761078 10.3322/canjclin.55.2.74

[4] Yang L, Parkin DM, Whelan S, Zhang S, Chen Y, et al (2005) Statistics on cancer in China: cancer registration in 2002. Eur J Cancer Prev 14: 329–35. 16030422 10.1097/00008469-200508000-00004

[5] Jemal A, Siegel R, Xu J, Ward E (2010) Cancer Statistics, 2010. CA Cancer J Clin 60: ; 277–300. 10.3322/caac.20073 20610543

[6] Peng H, Ma M, Han B (2011) Survival Analysis of 1,742 Patients with Stage IV Non-small Cell Lung Cancer. Zhongguo Fei Ai Za Zhi 14: 362–366. 21496437 10.3779/j.issn.1009-3419.2011.04.11 PMC5999708

[7] Kiessling R, Klein E, Pross H, Wigzell H (1975) ‘Natural’ killer cells in the mouse. II. Cytotoxic cells with specificity for mouse Moloney leukemia cells. Characteristics of the killer cell. Eur J Immunol 5: 117–121. 1086218 10.1002/eji.1830050209

[8] Kiessling R, Klein E, Wigzell H (1975) ‘Natural’ killer cells in the mouse. I. Cytotoxic cells with specificity for mouse Moloney leukemia cells. Specificity and distribution according to genotype. Eur J Immunol 5: 112–117. 1234049 10.1002/eji.1830050208

[9] Kim R, Emi M, Tanabe K (2007) Cancer immunoediting from immune surveilance to immune escape. Immunology 121: 1–14. 17386080 10.1111/j.1365-2567.2007.02587.x PMC2265921

[10] Vivier E, Ugolini S, Blaise D, Chabannon C, Brossay L (2012) Targeting natural killer cells and nat-ural killer T cells in cancer. Nat Rev Immunol 12; 239–252. 10.1038/nri3174 PMC5161343 22437937

[11] Coca S, Perez-Piqueras J, Martinez D, Colmenarejo A, Saez MA, et al. (1997) The prognostic significance of intratumoral natural killer cells in patients with colorectal carcinoma. Cancer 79: 2320–2328. 10.1002/(sici)1097-0142(19970615)79:12<2320::aid-cncr5>3.0.co;2-p 9191519

[12] Ishigami S, Natsugoe S, Tokuda K, Nakajo A, Xiangming C, et al. (2000) Clinical impact of intratumoral natural killer cell and dendritic cell infiltration in gastric cancer. Cancer Lett 159: 103–108. 10.1016/s0304-3835(00)00542-5 10974412

[13] Villegas FR, Coca S, Villarrubia VG, Jiménez R, Chillón MJ, et al. (2002) Prognostic significance of tumor infiltrating natural killer cells subset CD57 in patients with squamous cell lung cancer. Lung Cancer 35: 23–28. 10.1016/s0169-5002(01)00292-6 11750709

[14] Farag SS, Caligiuri MA (2006) Human natural killer cell development and biology. Blood Rev 20: 123–137. 16364519 10.1016/j.blre.2005.10.001

[15] Cooper MA, Fehniger TA, Turner SC, Chen KS, Ghaheri BA, et al (2001) Human natural killer cells: a unique innate immunoregulatory role for the CD56 (bright) subset. Blood 97: 3146–3151. 11342442 10.1182/blood.v97.10.3146

[16] Esendagli G, Bruderek K, Goldmann T, Busche A, Branscheid D, et al (2008) Malignant and non-malignant lung tissue areas are differentially populated by natural killer cells and regulatory T cells in non-small cell lung cancer. Lung Cancer 59: 32–40. 17825949 10.1016/j.lungcan.2007.07.022

[17] Zhou Q, Wang Y, Che G, Zhu W, Chen X, et al (2003) Establishment and their biological characteristics of clonal cell subpopulations (NL9980 and L9981) from a human lung large cell carcinoma cell line (WCQH-9801). Zhongguo Fei Ai Za Zhi 6: 464–468. 21310132 10.3779/j.issn.1009-3419.2003.06.13

[18] Ren Y, Chen J, Liu H, Yan H, Wang Y, et al (2008) The study of the tumorigenicity and metastasis ability in human lung cancer cell line L9981 using in vivo imaging. Zhongguo Fei Ai Za Zhi 11: 321–326. 20731927 10.3779/j.issn.1009-3419.2008.03.012

[19] Campoli M, Ferrone S (2008) Tumor escape mechanisms: potential role of soluble HLA antigens and NK cells activating ligands. Tissue Antigens 72: 321–334. 18700879 10.1111/j.1399-0039.2008.01106.x PMC2729103

[20] Zitvogel L, Tesniere A, Kroemer G (2006) Cancer despite immunosurveillance: immunoselection and immunosubversion. Nat Rev Immunol 6: 715–727. 16977338 10.1038/nri1936

[21] LeMaoult J, Zafaranloo K, Le Danff C, Carosella ED (2005) HLA-G up-regulates ILT2, ILT3, ILT4, and KIR2DL4 in antigen presenting cells, NK cells, and T cells. Faseb J 19: 662–664. 15670976 10.1096/fj.04-1617fje

[22] Levy EM, Bianchini M, Von Euw EM, Barrio MM, Bravo AI, et al (2008) Human leukocyte antigen-E protein is overexpressed in primary human colorectal cancer. Int J Oncol 32: 633–641. 18292941

[23] Urosevic M, Dummer R (2008) Human leukocyte antigen-G and cancer immunoediting. Cancer Res 68: 627–630. 18245459 10.1158/0008-5472.CAN-07-2704

[24] Salazar-Onfray F, Lopez MN, Mendoza-Naranjo A (2007) Paradoxical effects of cytokines in tumor immune surveillance and tumor immune escape. Cytokine Growth Factor Rev 18: 171–182. 17329145 10.1016/j.cytogfr.2007.01.015

[25] Castriconi R, Cantoni C, Della Chiesa M, Vitale M, Marcenaro E, et al (2003) Transforming growth factor beta 1 inhibits expression of NKp30 and NKG2D receptors: consequences for the NK-mediated killing of dendritic cells. Proc Natl Acad Sci U S A 100: 4120–4125. 12646700 10.1073/pnas.0730640100 PMC153058

[26] Richards JO, Chang X, Blaser BW, Caligiuri MA, Zheng P, et al (2006) Tumor growth impedes natural-killer-cell maturation in the bone marrow. Blood 108: 246–252. 16556890 10.1182/blood-2005-11-4535 PMC1895835

[27] Schleypen JS, Baur N, Kammerer R, Nelson PJ, Rohrmann K, et al (2006) Cytotoxic markers and frequency predict functional capacity of natural killer cells infiltrating renal cell carcinoma. Clin Cancer Res 12: 718–725. 16467081 10.1158/1078-0432.CCR-05-0857

[28] Schleypen JS, Von Geldern M, Weiss EH, Kotzias N, Rohrmann K, et al (2003) Renal cell carcinoma infiltrating natural killer cells express differential repertoires of activating and inhibitory receptors and are inhibited by specific HLA class I allotypes. Int J Cancer 106: 905–912. 12918068 10.1002/ijc.11321

[29] Carlsten M, Norell H, Bryceson YT, Poschke I, Schedvins K, et al (2009) Primary human tumor cells expressing CD155 impair tumor targeting by down-regulating DNAM-1 on NK cells. J Immunol 183: 4921–4930. 19801517 10.4049/jimmunol.0901226

[30] Dasgupta S, Bhattacharya-Chatterjee M, O'Malley BW Jr, Chatterjee SK (2005) Inhibition of NK Cell Activity through TGF-beta 1 by down-regulation of NKG2D in a murine model of head and neck cancer. J Immunol 175: 5541–5550. 16210663 10.4049/jimmunol.175.8.5541

[31] Hayakawa Y, Kelly JM, Westwood JA, Darcy PK, Diefenbach A, et al (2002) Cutting edge: tumor rejection mediated by NKG2D receptor-ligand interaction is dependent upon perforin. J Immunol 169: 5377–5381. 12421908 10.4049/jimmunol.169.10.5377

[32] Sullivan LC, Clements CS, Rossjohn J, Brooks AG (2008) The major histocompatibility complex class Ib molecule HLA-E at the interface between innate and adaptive immunity. Tissue Antigens 72: 415–424. 18946929 10.1111/j.1399-0039.2008.01138.x

[33] Ljunggren HG, Kärre K (1990) In search of the ‘missing self’: MHC molecules and NK cell recognition. Immunol Today 11: 237–244. 2201309 10.1016/0167-5699(90)90097-s

[34] Kärre K (2002) NK cells, MHC class I molecules and the missing self. Scand J Immunol 55: 221–228. 11940227 10.1046/j.1365-3083.2002.01053.x

[35] Peng Y, Luo G, Zhou J, Wang X, Hu J, et al (2013) CD86 is an activation receptor for NK cell cytotoxicity against tumor cells. PLoS One 8: e83913. 24349559 10.1371/journal.pone.0083913 PMC3859666

[36] Glasner A, Ghadially H, Gur C, Stanietsky N, Tsukerman P, et al (2012) Recognition and prevention of tumor metastasis by the NK receptor NKp46/NCR1. J Immunol 188: 2509–2515. 22308311 10.4049/jimmunol.1102461

